# Production of lentiviral vectors using novel, enzymatically produced, linear DNA

**DOI:** 10.1038/s41434-018-0056-1

**Published:** 2019-01-14

**Authors:** Rajvinder Karda, John R. Counsell, Kinga Karbowniczek, Lisa J. Caproni, John P. Tite, Simon N. Waddington

**Affiliations:** 1 Gene Transfer Technology Group, Institute for Women’s Health, University College London, London, UK; 2 Dubowitz Neuromuscular Centre, Molecular Neurosciences Section, Developmental Neurosciences Programme, UCL Great Ormond Street Institute of Child Health, London, UK; 3 NIHR Great Ormond Street Hospital Biomedical Research Centre, London, UK; 4 Touchlight Genetics Ltd, Hampton, UK; 5 SA/MRC Antiviral Gene Therapy Research Unit, Faculty of Health Sciences, University of the Witswatersrand, Johannesburg, South Africa

**Keywords:** Gene expression, Genetic vectors

## Abstract

The manufacture of large quantities of high-quality DNA is a major bottleneck in the production of viral vectors for gene therapy. Touchlight Genetics has developed a proprietary abiological technology that addresses the major issues in commercial DNA supply. The technology uses ‘rolling-circle’ amplification to produce large quantities of concatameric DNA that is then processed to create closed linear double-stranded DNA by enzymatic digestion. This novel form of DNA, Doggybone™ DNA (dbDNA™), is structurally distinct from plasmid DNA. Here we compare lentiviral vectors production from dbDNA™ and plasmid DNA. Lentiviral vectors were administered to neonatal mice via intracerebroventricular injection. Luciferase expression was quantified in conscious mice continually by whole-body bioluminescent imaging. We observed long-term luciferase expression using dbDNA™-derived vectors, which was comparable to plasmid-derived lentivirus vectors. Here we have demonstrated that functional lentiviral vectors can be produced using the novel dbDNA™ configuration for delivery in vitro and in vivo. Importantly, this could enable lentiviral vector packaging of complex DNA sequences that have previously been incompatible with bacterial propagation systems, as dbDNA™ technology could circumvent such restrictions through its phi29-based rolling-circle amplification.

## Introduction

Lentiviral vectors provide important advantages in gene therapy applications, such as a large transgene-carrying capacity [1] and the ability to transduce dividing and non-dividing cells [2]. Despite the development of packaging and producer cell lines, lentivirus vectors are predominantly manufactured by transient transfection of HEK293T cells with plasmid DNA (pDNA) cassettes encoding the vector genome, HIV-1 structural proteins and trans-acting factors. These pDNAs are required at a sizeable manufacturing scale and represent a significant bottleneck to flexible clinical programmes.

There are several drawbacks associated with bio-production of lentivirus vector titres on a pDNA platform. GMP pDNA manufacture is costly, complex and the DNA product is ultimately contaminated with bacterial propagation elements that are unnecessary for virus production in mammalian cells. Furthermore, eukaryotic expression cassettes may occasionally contain gene sequences that produce toxic or problematic effects in bacteria, which limits their amplification.

Touchlight’s novel, synthetic in vitro amplification process is capable of producing GMP DNA to multi-gram scale in 2 weeks (WO 2010/086626 5 August 2010). The process uses rolling-circle amplification by phi29 polymerase to produce long concatameric repeats of the template DNA [3]. These concatamers are subsequently reduced to individual closed linear DNA molecules called Doggybone™ DNA (dbDNA™) through the action of the protelomerase enzyme TelN [3]. The process is high-fidelity (1/10^6^–1/10^7^), highly processive (approximately 70 kbp) and has the added benefit of being capable of amplifying complex DNA sequences, without loss of fidelity or yield [4]. The dbDNA™ is minimal, containing only the user-defined sequences of interest, with no antibiotic resistance gene or origin of replication. This has implications for the induction of immune stimulation effects [5] both in vitro [6] and in vivo [7]. Thus dbDNA™ potentially offers a safer, more clinically relevant DNA construct for use in virus manufacturing. We have previously observed that, unlike pDNA containing bacterial elements, dbDNA™ does not evoke recognition by TLR9 [7–10].

Here we investigated whether lentivirus vectors could be produced using dbDNA™. We demonstrate that lentiviral vectors can be produced using all combinations of dbDNA™ and pDNA in a three-component production system. Importantly, we show that vectors derived solely using dbDNA™ constructs produce comparable transgene expression to plasmid-derived lentivirus vectors in vitro and titre-matched vectors demonstrated similar transgene expression in vivo.

## Results

### Synthesis of dbDNA™ lentivirus vector payload

dbDNA™ was synthesised, as previously described [9], using a proTLx-K template plasmid derived from a lentivirus backbone containing codon-optimised firefly luciferase under transcriptional regulation of the spleen focus-forming virus promoter (SFFV), linked to green fluorescent protein (GFP) by a 2A self-cleaving peptide (pLNT-SFFV-Luc-2A-GFP, Supplementary Figure 1A) [11].

In brief, the lentivirus transgene backbone from pLNT-SFFV-Luc-2A-GFP (including the long terminal repeats) was cloned into the Touchlight proTLx-K template plasmid in between the protelomerase telRL sites. This construct was amplified by rolling circle replication using *Bacillus subtilis* phage phi29 DNA polymerase, generating long concatamer repeats. *Escherichia coli* phage N15 protelomerase TelN was used to cleave and join double-stranded sequences containing a single cassette. Initial amplification used small amounts of pDNA as a template, which, following its selective degradation with restriction enzymes and exonucleases, was fully degraded, leaving dbDNA™ only. Simple size-based separation removed dNTPs, primer and enzymes while facilitating buffer exchange. This process takes 4–5 days and is then followed by an external quality control, which is 1 working week.

### In vivo transgene expression from payload dbDNA^TM^

To determine whether the payload dbDNA™ construct was capable of mediating gene expression, we delivered it in vivo by hydrodynamic injection into the tail vein of adult mice as previously described [12].

The following constructs were administered; two plasmid constructs, pLNT-SFFV-Luc-2A-GFP and proTLx-K SFFV-Luc-2A-GFP, and the doggybone construct db-SFFV-Luc-2A-GFP (Supplementary Figure 1). The proTLx-K SFFV-Luc-2A-GFP construct is a matched control to the dbDNA™ construct, containing the telR/L sequences flanking the gene expression cassette.

**Fig. 1:**
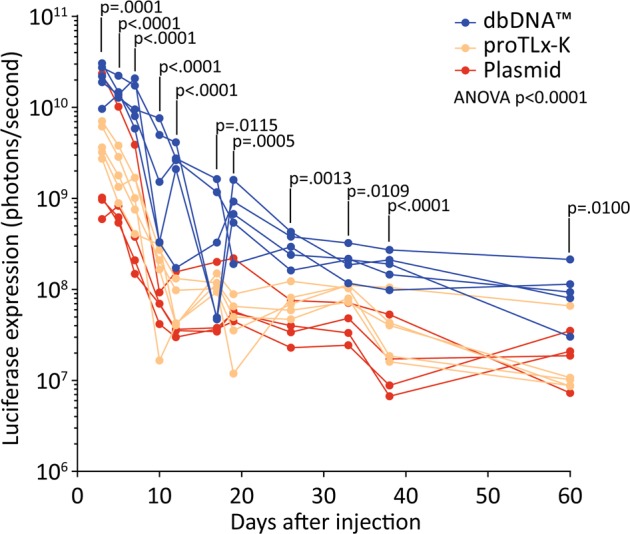
Luciferase expression quantified from Hydrodynamic injections of DNA. Plasmid, proTLx-K and dbDNA™ constructs were molarity matched and administered to 6-week-old mice by hydrodynamic injections via the tail vein (*n* = 5 for proTLx-K and dbDNA™ constructs and *n* = 4 for plasmid construct). The expression of luciferase was quantified post injection. Luciferase expression from dbDNA™ was consistently significantly higher than from plasmid DNA (repeated-measures analysis of variance with Dunnett’s post hoc test); luciferase expression from proTLx-K was similar to that from plasmid. Individual mice are plotted

Whole-body bioluminescence imaging revealed that luciferase expression from the dbDNA™ construct was significantly greater at each imaging time point than from the plasmid and proTLx-K payload constructs (Fig. 1).

### Vector production using different combinations of plasmid and dbDNA™

Lentiviral vectors were produced using different combinations of dbDNA™ and pDNA encoding payload, envelope and packaging sequences to determine whether components required for vector production could be supplied by using the dbDNA™ constructs. The VSV-G envelope protein used in this vector has a broad tissue tropism [13].

Each lentiviral vector was quantified by three methods: p24 enzyme-linked immunosorbent assay (ELISA), fluorescence activated cell sorting (FACS) analysis, and quantitative polymerase chain reaction (qPCR). p24 ELISA reveals the total number of viral particles per ml, FACS analysis measures the number of transducing units per ml, and qPCR detects the reverse-transcribed viral genome copy number per ml. The plasmid templates (proTLx-K constructs) that were used to synthesise the dbDNA™ were also used to produce lentiviral vectors as a control (Supplementary Table 1B), as these constructs not only contain the same sequence as the dbDNA™ constructs but also have all the elements required for plasmid propagation in bacteria.

**Fig. 2:**
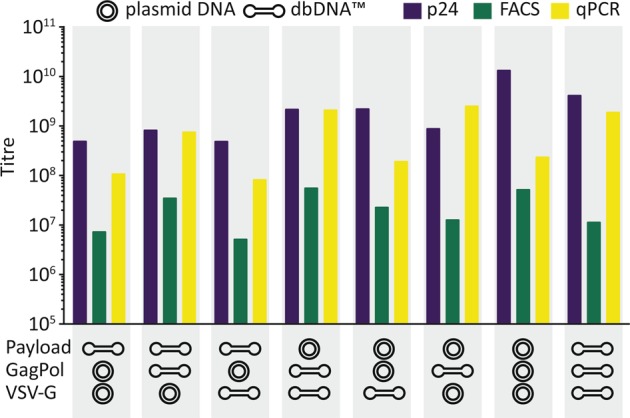
Summary of titration values for all lentiviral vectors produced. Lentiviral vectors using a combination of plasmid and dbDNA™ (payload, packaging and envelope DNA) constructs were titred by p24, fluorescence activated cell sorting and quantitative polymerase chain reaction analysis

**Fig. 3:**
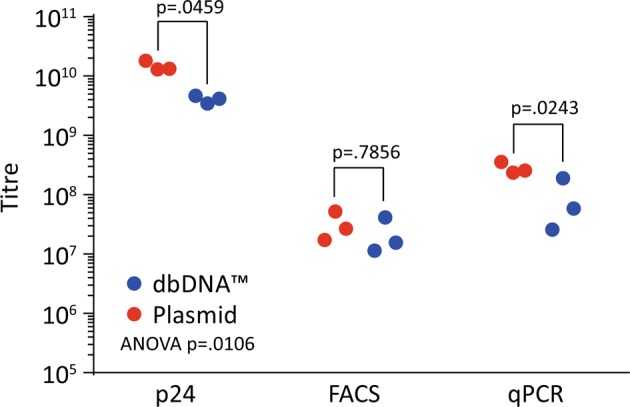
Comparison of titres between lentiviral vectors produced from plasmid and dbDNA™. Lentiviral vector produced from plasmid had a significantly higher titre than that produced from dbDNA™ when titred using p24 and quantitative polymerase chain reaction assay but not by fluorescence activated cell sorting (two-way analysis of variance with Sidak’s post hoc test)

We demonstrate that lentiviral vectors could be produced using all combinations of dbDNA™ and comparable titres were obtained for each DNA format (six separate vector batches) (Fig. 2). Figure 3 shows the titres achieved in three separate experiments. The results revealed that both sets of constructs generated good titres of lentiviral vector. The titres of the dbDNA™ encoded vectors were significantly lower than the plasmid equivalents in the p24 assay (*P* < 0.001) but there was no difference by FACS or qPCR.

### In vitro comparison of transgene expression from pDNA or dbDNA™ vectors

**Fig. 4:**
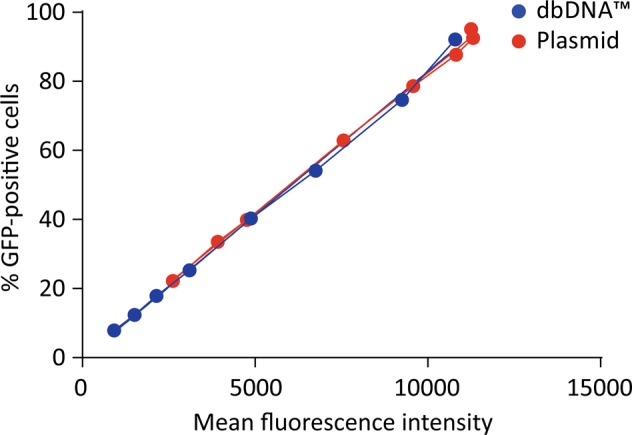
In vitro comparison of dbDNA™ and plasmid lentiviral vectors. Mean fluorescence intensity correlated equally with green fluorescent protein (GFP)-positive cells for vector generated by both dbDNA™ and plasmid vector

To examine the transduction efficiencies of lentiviral vectors produced by either plasmid or dbDNA™, a dose escalation of each vector was applied to HEK 293T cells. Seventy-two hours after transduction, FACS analysis was conducted, revealing that plasmid and dbDNA™ lentiviral vectors produced a similar dose–response, as seen by plotting the percentage of GFP-positive cells vs the mean fluorescence intensity (MFI) (Fig. 4). However, comparing the lowest 3 doses, we observed that plasmid lentiviral vectors give rise to ~2-fold higher percentage of GFP-positive cells and MFI than dbDNA™ lentiviral vectors.

### In vivo validation of titred matched plasmid vs dbDNA™ lentiviral vectors

**Fig. 5:**
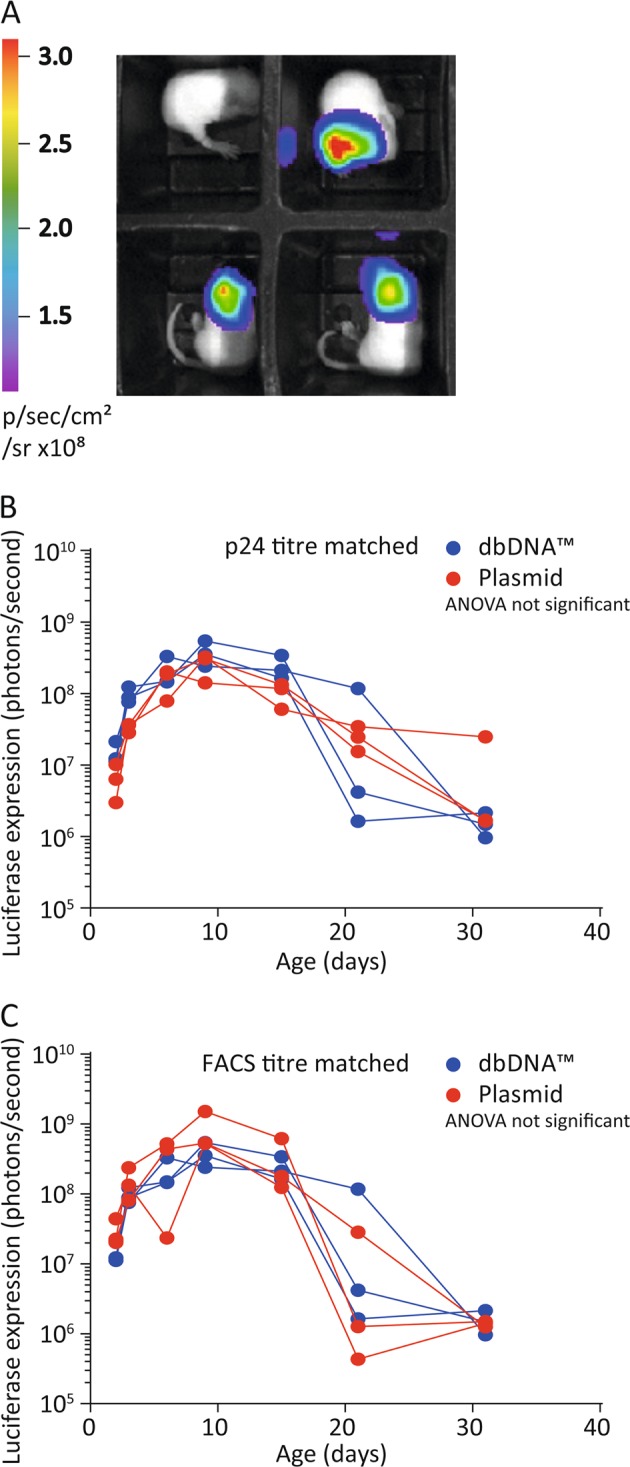
Whole-body bioluminescence imaging of titre-matched plasmid and dbDNA™ vector. **a** Plasmid and dbDNA™ lentiviral vector were titred matched by either p24 or fluorescence activated cell sorting (FACS) data. The vectors were administered to neonatal mice via intracranial administration (*n* = 3 for each vector). Whole-body bioluminescence images were taken every other day. There was no significant difference in bioluminescence following injection of either **b** p24 titre-matched vectors or **c** FACS titre-matched vectors. Bioluminescence from individual mice is plotted

Neonatal mice received a dose-matched intracerebroventricular injections of lentiviral vectors produced by either dbDNA™ or plasmid templates, with doses matched by either p24 titre (viral particles) or FACS titre (transducing units). The vectors were administered to P1 neonatal mice by intracranial injection, as described previously [14]. Luciferase expression was quantified by whole-body bioluminescence imaging over the course of development (Fig. 5a).

There was no significant difference in luciferase expression between the two vectors in vivo with p24 matched virus, *P* = 0.43, 3 mice per group (Fig. 5b). We repeated this experiment with vector titres matched as per FACS titre where, again, we observed no significant difference in luciferase expression *P* = 0.33, 3 mice per group (Fig. 5c).

## Discussion

In this report, we have described the ability of dbDNA™ constructs to generate functional lentiviral vectors with comparable in vivo transduction efficiency to those produced with plasmid constructs. From envelope, packaging and payload templates, it was possible to generate 1.5 mg/ml of payload, 2.6 mg/ml of envelope and 1.5 mg/ml of packaging DNA.

Hydrodynamic injection of pDNA was first reported by Liu et al. [15]. Here we observed significantly higher expression from dbDNA™ after a single injection, approximately 6-fold greater than pDNA 8 weeks after injection. This is consistent with the work of Lu and colleagues [16], where transcriptional silencing was observed with constructs containing >1 kb of DNA outside of the expression cassette. In our dbDNA™ constructs, 6.9 kb exists between the expression cassette and the 5’ and 3’ loops, respectively. An alternative explanation that TelN sequences confer persistence of expression can be disregarded, since proTLx-K plasmid does result in expression significantly higher than the original payload plasmid. Indeed, these data warrant further investigation into the mechanism for enhanced dbDNA™ expression in vivo, in future studies.

The primary aim of our experiment was to validate expression from the dbDNA™ payload construct. Importantly, our data demonstrate that production of functional lentiviral vectors using dbDNA™ can be achieved, with transgene expression levels comparable to those of plasmid-derived vectors.

We consequently tested these vectors in vivo by titre-matching the plasmid and dbDNA™ lentivirus based on p24 and FACS titration methods. The vectors were administered to neonatal mice via intracerebroventricular injections and luciferase expression was monitored over the course of development. These novel findings revealed a similar trend in expression from both plasmid and dbDNA™ titre-matched lentiviral vectors. Although vector generated from pDNA was significantly higher than that generated by dbDNA™, it is important to note that we did not optimise PEI:DNA ratios for dbDNA™.

The use of an enzymatic DNA amplification platform to produce constructs for lentivirus production has a number of potential key advantages, namely speed of production, cost of goods, elimination of bacterial sequences (such as origins of replication and antibiotic selection markers) and avoidance of carry-over of bacterial contaminants (such as lipopolysaccharide). However, a key advantage of dbDNA™ arises in its ability to amplify sequences that are difficult to propagate in bacteria, due to sequence toxicity or complexity. A number of therapeutically meaningful transgenes have been identified as being toxic to bacteria, including some sodium ion channels [17] and calcium channels [18]. The use of an enzymatic DNA amplification technology might therefore provide an important platform for the investigation and therapeutic development of candidates for diseases related to these transgenes. It would be useful to interrogate whether there is any difference in inflammatory and immune markers between plasmid- and dbDNA™-produced lentivirus vectors. We conducted the in vivo validation using neonatal mice, which, unlike adult mice, develop immune tolerance to vectorised transgenes [19, 20] and are immunologically ignorant to vector components [21]. It would be interesting to analyse the potential benefits for reduced inflammatory and immune markers in adult animals in future studies.

In summary, in this study we were able to show for the first time the production of lentiviral vectors using DNA constructs produced by enzymatic amplification. The in vitro and in vivo readouts were comparable with our traditional plasmid-produced lentiviral vectors, despite the utilisation of systems not optimised for the differing topology of dbDNA™ compared to plasmid. This platform could represent a significant advance for the development of gene therapies for previously intractable diseases limited by difficulty in manipulation of pDNA.

## Material and methods

### dbDNA™ production

Required sequences of the lentivirus payload, envelope and packaging plasmids were sub-cloned into the proTLx-K template plasmid inside the protelomerase telR/L sequences. Verification of correct clones was done by Sanger sequencing.

The template plasmids were denatured using 0.1 M NaOH for 5 min at 30 °C followed by quenching in reaction buffer (30 mM Tris–HCl pH 7.4, 5 mM (NH_4_)_2_SO_4_, 30 mM KCl, 7.5 mM MgCl_2_, 2 mM dithiothreitol) containing 50 μM custom primer (Metabion, Germany), 4 mM dNTPs (Bioline, London, UK), 200 units/ml Phi29 polymerase (Enzymatics, Manchester, UK) and 0.05 units/ml pyrophosphatase (Enzymatics). The reaction was incubated at 30 °C for 30 h and the resulting concatameric DNA processed by addition of 2 mM TelN protelomerase (Enzymatics). Additional processing was performed using 200 units/ml restriction enzyme suitable for plasmid backbone cleavage (template dependent) (Enzymatics or New England Biolabs, Hitchin, UK) and 200 units/ml exonuclease ExoIII (Enzymatics). The dbDNA™ was purified from the reaction components by addition of 500 mM NaCl/100 mM MgCl_2_ and precipitated using 3.9% polyethylene glycol (PEG) 8000 (Applichem, Bredbury, UK). dbDNA™ was pelleted (10 min, 4500 g) and re-suspended in 20 ml 500 mM NaCl/100 mM MgCl_2_. The 3.9% PEG 8000 precipitation was repeated with final resuspension in water prior to ethanol precipitation to remove residual salts.

### Lentiviral vector production

HEK293T cells for viral production were seeded overnight in a T175-cm^2^ flask at 2 × 10^7^ cells. Cells were transfected with 50 µg of payload plasmid or dbDNA^TM^, 17.5 µg of pMD.G2 (VSV-G envelope plasmids, or VSV-G dbDNA^TM^) and 32.5 µg of pCMVΔR8.74 (packaging plasmid and dbDNA^TM^) with 1 µl of polyethylenimine (10 mM) (Sigma-Aldrich, Dorset, UK) in 12 ml of Optimem for 3 h. Media was replaced with fresh Dulbecco’s modified Eagle’s medium supplemented with 10% fetal calf serum. Forty-eight hours after transfection, vector supernatant was filter sterilised (0.22 µm) and concentrated by centrifugation at 5000 rpm for 20 h. The centrifuged viral vector pellet was resuspended with Optimem and stored at −80 °C.

### Titration of vectors

HEK293T cells (2.5 × 10^5^) were seeded into a 96-well plate. The detection of the enhanced GFP (eGFP) was undertaken using a BD Verse flow cytometer. eGFP fluorescence was excited using a 488 nm laser. Analysis of flow cytometry was conducted as previously described [22]. All flow cytometric data were analysed by the FlowJo software version 9.3.1 (Tree Star, Inc).

HEK293T cells (2.5 × 10^5^) were plated into a 24-well plate and transduced with a range of volumes of concentrated lentivirus. Seventy-two hours after transduction, genomic DNA was extracted and the provirus titre calculated by qPCR, as described previously [23]. The viral capsid number was determined using a Lenti-X^TM^ p24 Rapid Titer Kit (Takara, Saint-Germain-en-Laye, France). The capsid number was determined according to the kit manufacturer’s calculations and previously described in Vink et al. [22].

### Animal procedures

Outbred CD1 mice used in this study were supplied by Charles Rivers. All animal experiments conducted within this study were in agreement with the United Kingdom Home Office guidelines, approved by the ethical review committee and following the institutional guidelines at University College London.

### Hydrodynamic tail vein injections

Male CD1 mice of 6 weeks of age were used. Mice were placed in a chamber at 32 °C for 10 min. They were anaesthetised with isoflurane with 100% oxygen (Abbott Laboratories, London, UK). DNA amounts of 10 µg of plasmid and proTLx DNA and 7.8 µg of dbDNA™ (to ensure equal molarity of DNA) made up to 2 ml of phosphate-buffered saline were injected over approximately 5 s into adult mice.

### Neonatal intracerebroventricular injections

For intracerebroventricular injections on the day of birth, mice were subjected to brief hypothermic anaesthesia followed by unilateral injections of concentrated lentiviral vector (5 µl in total) into the cerebral lateral ventricles using a 33-gauge Hamilton needle (Fisher Scientific, Loughborough), following co-ordinates provided by Kim et al. [24].

### Whole-body bioluminescence imaging

Where appropriate, mice were anaesthetised with isoflurane with 100% oxygen and/or received an intraperitoneal injection of 15 mg/ml of D-luciferin (Gold Biotechnologies, St. Louis, USA). Mice were imaged after 5 min using a cooled charged-coupled device camera (IVIS machine, Perkin Elmer, Coventry, UK) between 1 s and 5 min. The regions of interest were measured using the Living Image Software (Perkin Elmer) and expressed as photons per second per centimetre squared per steradian (photons/second/cm^2^/sr).

### Statistics

The exact sample sizes of each experimental group/condition are detailed in the respective legends of each figure. The number of replicates is stated in the respective section of Results. Where possible, two-way analysis of variance (ANOVA) with the appropriate post hoc test was performed, using GraphPad Prism. ANOVA has been shown to be robust to non-normal data. Individual plots are provided for each animal. Exact *P* values are stated except when the overall test is not significant. The number of replicates was the minimum possible to address our hypothesis of whether it was possible to produce lentivirus vectors using dbDNA™ and if this vector was equivalent, both in vitro and in vivo, to vector produced using pDNA. No power calculations were performed. All injected animals were included in the published analysis. Mice were selected randomly for injection. Imaging was not performed blind.

## Supplementary information

Supplementary Figure and Table
