# Supplementary material for: Production of lentiviral vectors using novel, enzymatically produced, linear DNA
Source: Gene Ther. 2019 Jan 14;26(3):86–92. doi: 10.1038/s41434-018-0056-1 (PMC6760675; doi:10.1038/s41434-018-0056-1)
Supplement: Supplementary file 1 — Supplementary Figure and Table [file 41434_2018_56_MOESM1_ESM.pdf]

Production of lentiviral vectors using novel, enzymatically-produced, linear DNA vectors

Rajvinder Karda[1], John R Counsell[2,3], Kinga Karbowniczek[4], Lisa J Caproni[4], John P Tite[4], Simon N Waddington[1,5]

1. Gene Transfer Technology Group, Institute for Women's Health, University College London, UK
2. Dubowitz Neuromuscular Centre, Molecular Neurosciences Section, Developmental Neurosciences Programme, UCL Great Ormond Street Institute of Child Health, London, UK.
3. NIHR Great Ormond Street Hospital Biomedical Research Centre, London, UK
4. Touchlight Genetics Ltd, Hampton, UK
5. SA/MRC Antiviral Gene Therapy Research Unit, Faculty of Health Sciences, University the Witswatersrand, Johannesburg, South Africa

A

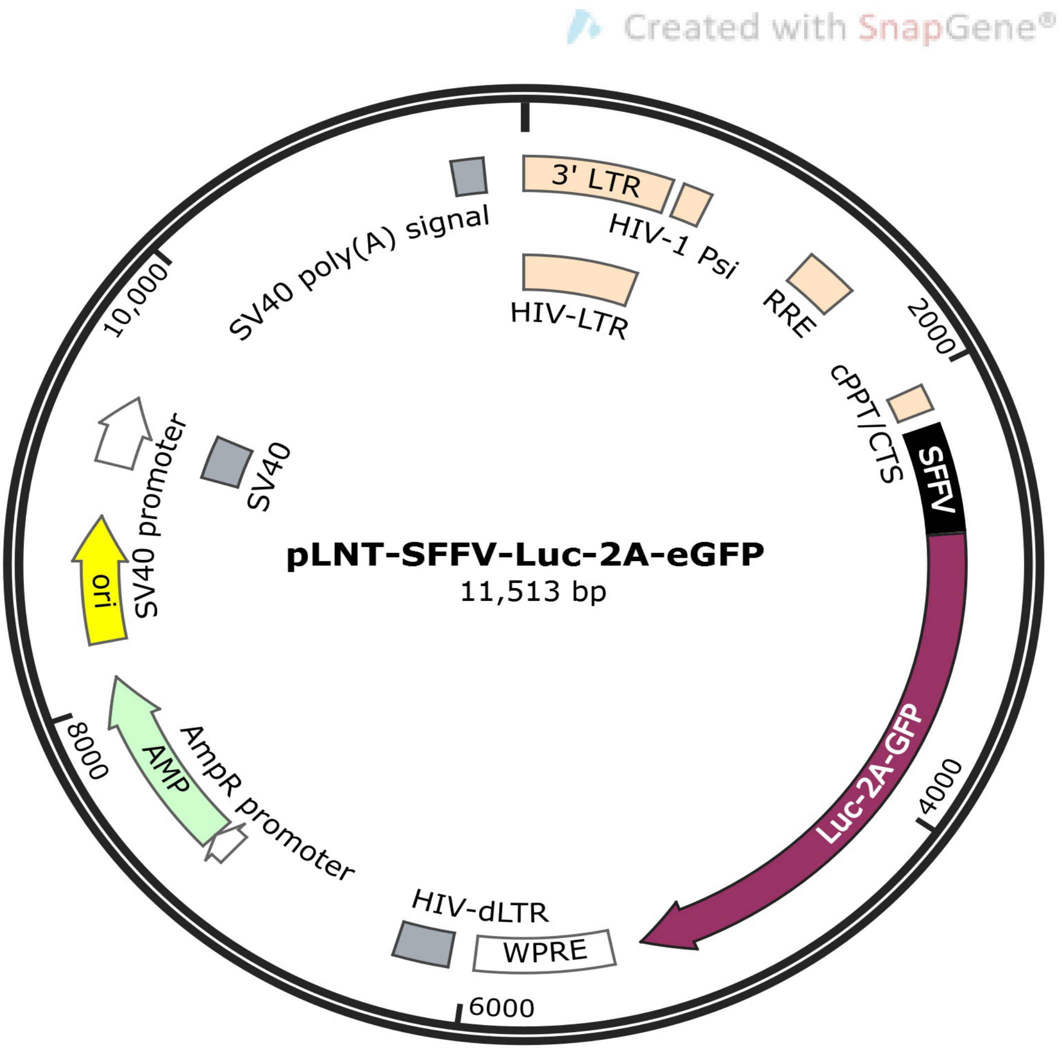

B

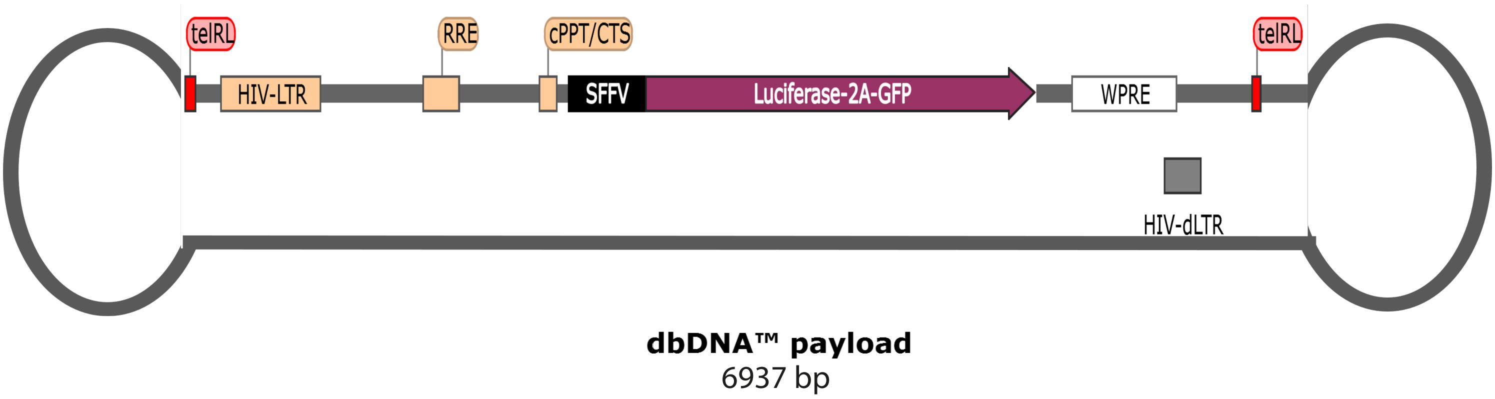

Supplementary Figure 1 – Schematic diagram of constructs. (A) pLNT-SFFV-Luc-2A-GFP contains a constitutive SFFV promoter, driving a codon-optimised firefly luciferase, linked by a bicistronic linker to GFP. These elements are flanked by a 3' and 5' Long terminal repeats (LTR). **(B)** The dbDNA™ payload contains all the features mentioned above, without the bacterial components. dbDNA™ is capped inside the telR/L sequences. Constructs produced using SnapGene®.

A

Lentivirus vectors	p24	FACS	qPCR
dbDNA™ pay, pDNA pack and env	4.93E+08	7.30E+06	1.08E+08
dbDNA™ env, pDNA pack and pay	2.21E+09	2.28E+07	1.92E+08
dbDNA™ pack, pDNA pay and env	8.84E+08	1.27E+07	2.52E+09
dbDNA™ pay and pack, pDNA env	8.23E+08	3.49E+07	7.57E+08
dbDNA™ pay and env, pDNA pack	4.90E+08	5.16E+06	8.27E+07
dbDNA™ pack and env, pDNA pay	2.18E+09	5.59E+07	2.11E+09
dbDNA™ pay, pack and env	4.15E+09	1.14E+07	1.89E+09
pDNA pay, pack and env	1.30E+10	1.72E+07	3.57E+08

B

Lentivirus vectors	p24	FACS	qPCR
pDNA pay, pack and env batch 1	1.30E+10	1.72E+07	3.75E+09
pDNA pay, pack and env batch 2	1.81E+10	2.67E+07	2.56E+08
pDNA pay, pack and env batch 3	1.33E+10	5.21E+07	2.36E+08
dbDNA™ pay, pack and env batch 1	4.15E+09	1.14E+07	1.89E+07
dbDNA™ pay, pack and env batch 2	4.67E+09	1.55E+07	2.60E+07
dbDNA™ pay, pack and env batch 3	3.45E+09	4.13E+07	5.85E+07
proTLx pay, pack and env batch 1	2.38E+10	3.15E+07	3.10E+10
proTLx pay, pack and env batch 2	2.18E+10	5.22E+06	8.24E+09
proTLx pay, pack and env batch 3	2.16E+10	3.63E+07	1.02E+10

Supplementary Table 1 – Titre values for all lentivectors produced. (A) p24, FACS and qPCR titres of all lentiviral vectors produced using combinations of plasmid and dbDNA™. **(B)** Titres of three separate batches of vectors produced using either plasmid, dbDNA™ and proTLx constructs.
